# Supplementary material for: Predicting the Stability of Organic Matter Originating from Different Waste Treatment Procedures
Source: Int J Environ Res Public Health. 2023 Jan 25;20(3):2151. doi: 10.3390/ijerph20032151 (PMC9916240; doi:10.3390/ijerph20032151)
Supplement: Supplementary file 1 [file ijerph-20-02151-s001.zip › Figure S1 Fluorescence regionalization integration for spectra interpretation and quantification.pdf]

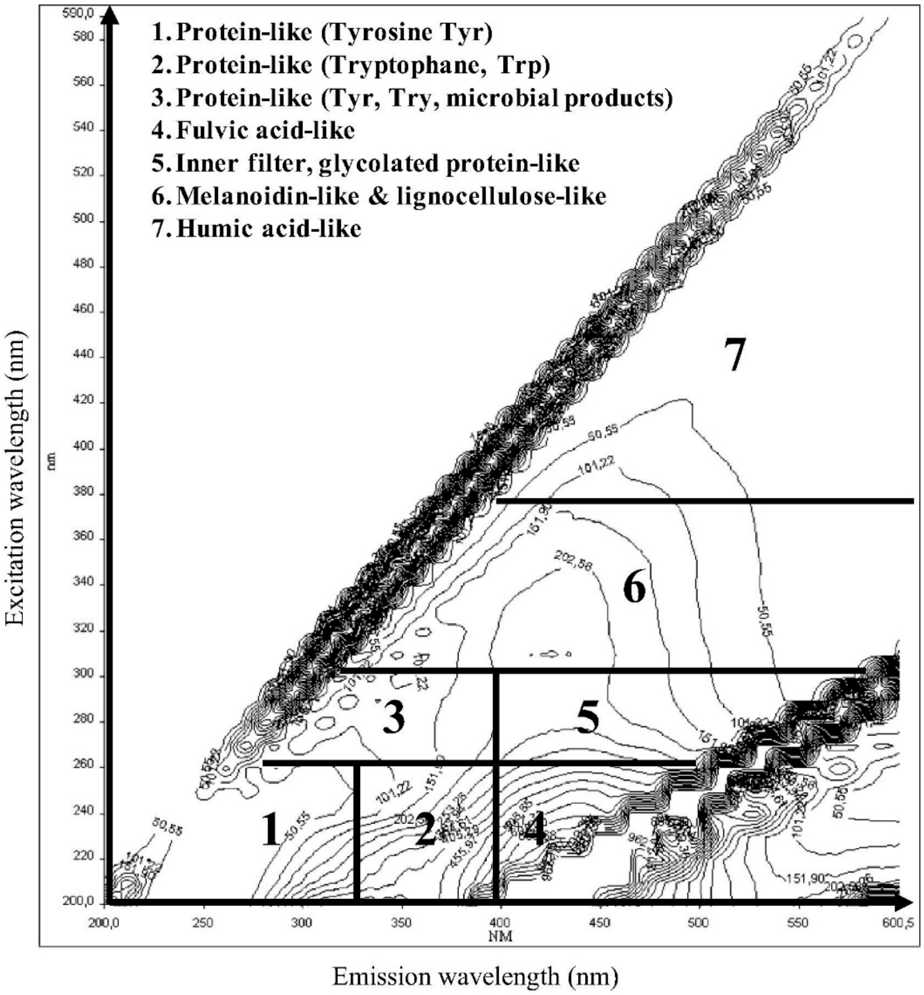

Figure S1. Fluorescence regionalization integration for spectra interpretation and quantification, x axis = emission wavelength (nm), y axis = excitation wavelength (nm) (Aemig et al., 2016)

Aemig, Q., Cheron, C., Delgenes, N., Jimenez, J., Houot, S., Steyer, J.-P., Patureau, D. 2016. Distribution of Polycyclic Aromatic Hydrocarbons (PAHs) in sludge organic matter pools as a driving force of their fate during anaerobic digestion. *Waste Management*, **48**, 389-396.
