# Supplementary material for: Predicting the Stability of Organic Matter Originating from Different Waste Treatment Procedures
Source: Int J Environ Res Public Health. 2023 Jan 25;20(3):2151. doi: 10.3390/ijerph20032151 (PMC9916240; doi:10.3390/ijerph20032151)
Supplement: Supplementary file 1 [file ijerph-20-02151-s001.zip › Table S1 Incubated samples and Cnm of OM in samples.pdf]

Table S1. C_{nm} (%) of OM in incubated samples

Sample	Manure1	Manure1-D	Manure1-C	Manure1-D-C	Biowaste2-D	Biowase2-C	Biowaste2-D-C1
C_{nm} (%)	50.9 ±1.9	67.2 ±3.0	81.6 ±2.5	83.6 ±3.6	75.9 ±1.9	75.7 ±1.9	80.2 ±1.7
Sample	Biowaste2-D-C2	Biowaste1-D	Biowaste1-C	Biowaste1-D-C1	Biowaste1-D-C2	Vetch1	Clover1
C_{nm} (%)	83.3 ±1.4	75.0 ±0.8	82.2 ±0.9	87.8 ±2.6	94.3 ±0.6	32.4 ±0.9	31.5 ±1.1
Sample	Stalk2	Mix5	Mix5-C3	Mix6	Mix6-C3	Mix1	Mix1-C3
C_{nm} (%)	37.8 ±0.6	64.9 ±0.1	69.2 ±0.6	60.2 ±1.6	76.5 ±0.6	69.8 ±0.1	87.0 ±0.3
Sample	Mix2	Mix2-C3	Mix3	Mix3-C3	Mix4	Mix4-C3	Stalk1-D
C_{nm} (%)	67.5 ±0.1	80.1 ±0.3	72.8 ±0.1	82.6 ±0.3	71.5 ±0.1	82.5 ±0.6	60.0
Sample	Biowaste3-D	Biowaste4-D	Biowaste5-D	Mix10-D1	Mix10-D2	Mix10-D3	Sludge2-D
C_{nm} (%)	68.0±0.3	81.0±1.1	73.4±0.9	64.3±1.3	68.7±0.5	62.7±1.6	60.8±0.9
